# Self-Help-Friendly Hospitals: Integrating Self-Help Groups as a Complementary Service in Healthcare

**DOI:** 10.3390/ijerph23040503

**Published:** 2026-04-14

**Authors:** Suzanne Lischer, Manuela Eder, Elina Lehmann, Oliver Kessler

**Affiliations:** 1 Lucerne School of Social Work, Lucerne University of Applied Sciences and Arts, 6002 Luzern, Switzerland; manuela.eder@stud.unilu.ch (M.E.); elina.lehmann@hslu.ch (E.L.); 2 Lucerne School of Business, Lucerne University of Applied Sciences and Arts, 6002 Luzern, Switzerland; oliver.kessler@hslu.ch

**Keywords:** self-help groups, patient-centered care, self-help friendliness, interprofessional collaboration, participation, healthcare

## Abstract

**Highlights:**

**Public health relevance—How does this work relate to a public health issue?**

- This work strengthens participatory structures and a self-help-friendly culture within hospitals to support patient-centered care.
- It recognizes self-help groups as a complementary component of healthcare services.

**Public health significance—Why is this work of significance to public health?**

- This work can lead to structural access to self-help resources for patients and within hospital settings.
- It facilitates strengthened, institutionalized cooperation between hospitals, self-help centers, and self-help groups as a basis for patient-centered care.

**Public health implications—What are the key implications or messages for practitioners, policy makers and/or researchers in public health?**

- There is a need to better include self-help groups within the healthcare system.
- Stable institutional support and interprofessional collaboration are important to maintain self-help structures and self-help-friendly approaches.

**Abstract:**

Background: This study reports the results of a project evaluation (2021–2024) that aimed to integrate self-help friendliness within the Swiss healthcare system and foster collaboration between hospitals, self-help centers, and self-help groups (SHGs). Methods: A mixed-methods design was used, comprising document analysis, standardized online surveys of hospital staff involved in 56 cooperative projects at two time points with repeated group comparisons (T0: *n* = 530, T1: *n* = 281), and in-depth case studies at four cooperation sites. Results: The descriptive findings indicate that the implemented measures contributed to achieving project objectives. Hospital staff increasingly perceived SHGs as complementary to professional care and reported more frequent provision of information to patients and their relatives. In addition, SHGs became more visible within hospitals. Conclusions: This study provides descriptive insights into the implementation of self-help friendliness in practice and suggests that introducing self-help friendliness in hospitals is both feasible and beneficial. Long-term, sustainable collaboration requires clear roles and responsibilities, organizational support, and recognition of the experiential expertise of SHGs.

## 1. Introduction

Self-help encompasses various practices that promote mutual support and problem-solving by patients and relatives, such as individual networking, local and virtual groups, peer support initiatives, and self-help organizations. All approaches of self-help share the principle that people facing similar challenges or life situations come together to support each other. Self-help groups (SHGs) differ from individual self-help and professionally guided groups by emphasizing peer-led interaction and shared experiences [1]. Alfred H. Katz [2] defines SHGs as “voluntary, small group structures for mutual aid and the accomplishment of a special purpose” (p. 135). These groups are typically formed by peers who seek support for shared challenges while fostering personal or social change. Therefore, self-help is based on mutual experience and support, taking place in non-bureaucratic settings without professional involvement [3]. They are typically member-led, though professionals may assist in the initial stages of their development. While traditionally grounded in face-to-face interaction, online SHGs have expanded substantially in recent years [4].

While acknowledging the existence of SHGs that address broader life crises, such as bereavement, family conflicts, or experiences of victimization [5], the following contribution focuses on health-related SHGs, which adopt an experience-based approach to managing health issues grounded in mutual exchange.

In addition to the potential benefits for patients and their relatives, SHGs can contribute to improved quality in the healthcare system. A qualitative survey of 45 SHGs showed that they provide emotional, practical, informational, and educational support through discussion groups, patient training, visitation services, and preventive outreach. Beyond these supportive functions, SHGs may also offer healthcare institutions valuable feedback and highlight deficiencies in interactions with medical settings, representing patient interests while also collaborating with key stakeholders across the healthcare system and the wider public [6]. However, not all patients or relatives participate in SHGs, and those who do are self-selected and participate voluntarily. Therefore, the perspectives shared within SHGs do not automatically reflect the broader patient population. Nevertheless, healthcare institutions can recognize SHGs as partners by engaging them in consultations, advisory panels, or co-design initiatives. Such engagement enables health professionals to incorporate patient perspectives into decision-making processes, thereby putting the collaborative principles of patient-centered care into practice.

Collaboration between patients, their relatives and caregivers, and healthcare professionals reflects a broader commitment to participatory and co-produced care, recognizing that patients and their relatives are typically laypersons without formal medical training. It is a core component of patient-centered care [7], aiming to integrate patients’ preferences, values, and beliefs into treatment and care processes while promoting their active involvement in decision-making and their empowerment within healthcare [8,9]. Within this broader framework, the recognition and inclusion of SHGs into the healthcare system can be understood as an organizational strategy for including patient participation and their experiential knowledge in healthcare institutions [7].

### 1.1. The Development of Self-Help Friendliness in Hospitals

The concept of self-help friendliness in hospitals emerged in the early 2000s as an effort to integrate SHGs more systematically into healthcare institutions. Early initiatives focused on cooperative models, such as coordinated information exchange [10], highlighting both the potential and the limitations of arrangements based primarily on an informal commitment. Building on these developments, the concept of self-help friendliness emerged as a framework for embedding collaboration with SHGs systematically within healthcare institutions, particularly hospitals. The concept refers to the extent to which institutions establish the structural, organizational, and cultural conditions needed to support the sustained integration of SHGs. It aims to create reliable conditions and frameworks for collaboration and operationalize patient-centered care by systematically incorporating the perspectives and resources of patients into healthcare delivery [11,12]. In this way, self-help friendliness contributes to the development of sustainable structures for integrating self-help practices into the healthcare system [13].

The self-help friendliness framework in hospitals thus operationalizes patient involvement in a structured yet flexible manner, balancing expert knowledge, patients’ and relatives’ needs, and patient influence. It represents a partnership model grounded in shared responsibility and mutual learning, aligning with contemporary concepts of co-produced health governance and service delivery [14]. Through this structure, the self-help friendliness framework not only formalizes collaboration but also addresses the shortcomings of informal approaches, ensuring that patient expertise and professional authority are meaningfully integrated into healthcare decision-making processes [15].

### 1.2. The Implementation of the Self-Help-Friendly Approach in Switzerland

In Switzerland, community-based self-help covers a wide range of topics and is supported by a multi-level organizational structure. Over 2700 SHGs address approximately 300 different topics, about three-quarters of which are health-related. These SHGs are usually organized informally or as registered associations and are not government entities [16]. Additionally, more than 200 specialized self-help organizations are active, with many offering group meetings in multiple locations. It is estimated that 65,000 individuals actively participate in these groups. Support is provided to these SHGs by 22 regional self-help centers, which promote, coordinate, and connect SHGs across social and health-related fields within their respective regions [1]. These centers are run by professional advisors who provide information and support, facilitate the development of local SHGs, and serve as regional points of contact. Staff of self-help centers understand their role primarily as supporting the establishment and ongoing functioning of SHGs [17]. Since 2000, the foundation Self-Help Switzerland has served as the national umbrella organization, promoting self-help across a wide range of issues and acting as a central coordination and service platform for regional self-help centers [15]. 

Despite the extensive network of SHGs and the central coordination by Self-Help Switzerland, SHGs were not systematically integrated into hospital care processes in Switzerland prior to the project presented in this article. Formal structures for collaboration between hospitals and SHGs were largely absent. To address this gap, the project “Health Literacy through Self-Help-Friendly Hospitals” (hereinafter referred to as “project”) was launched in 2021 as a national initiative by Self-Help Switzerland [18].

The project aimed to strengthen awareness among healthcare professionals, patients, and their relatives that self-help represents a complementary service to hospitalization and aftercare. Its objectives were to promote mutual self-help within hospital-related patient pathways, foster cooperation between hospitals, self-help centers, and SHGs across Switzerland, and examine how SHGs can be sustainably embedded within hospital structures.

If a hospital decides to participate in the project, a cooperation agreement is signed between Self-Help Switzerland and the hospital. Subsequently, cooperation teams are established—a collaborative triad consisting of professionals from regional self-help centers, the hospital’s self-help coordinator, and representatives of SHGs. These cooperation teams are responsible for defining and implementing the concrete measures that enable collaboration between SHGs, hospitals, and self-help centers within the larger project framework. This cooperation team structure is formalized through binding agreements that reflect the strategic commitment of healthcare institutions to promote a self-help friendliness framework [19]. Guided by six predefined quality criteria developed by Self-Help Switzerland and derived from the self-help friendliness framework developed in Germany [11], the cooperative teams jointly develop a comprehensive catalog of measures: a document that contains the actions developed within the cooperation triangle. It specifies how the healthcare institution implements these measures and documents the distribution of tasks and responsibilities between the healthcare institution, SHGs, and the self-help center [20]. These measures are organized around six quality criteria: (Q1) SHGs are enabled to present themselves within the hospital; (Q2) information about possible participation in SHGs is provided to the patients at the appropriate time; (Q3) the cooperation between SHGs and the hospital is communicated; (Q4) a designated contact person within the hospital is ensured; (Q5) information and experience exchange between SHGs, the self-help center, and the hospital is facilitated; and (Q6) active participation in SHGs is enabled [20].

Hospitals that successfully implement these measures and meet the six quality criteria may apply to Self-Help Switzerland and be awarded the “Self-Help Friendliness” label. The certificate is valid for two years and may be renewed. For this purpose, hospitals must resubmit documents as part of a standardized award renewal process, which Self-Help Switzerland then reviews.

The initial phase of the project lasted from 2021 to 2025. Following this period, Self-Help Switzerland decided to continue the framework as a model, securing additional funding sources and institutionalizing the project with minor conceptual modifications [19]. The evaluation presented in this article focuses on a 4-year period (2021–2024) [18].

### 1.3. Rationale and Objectives of the Study

On behalf of Health Promotion Switzerland, which funded the project and commissioned its evaluation, we monitored and assessed the project. The evaluation focused on organizational and process-related changes within the participating healthcare institutions, on whether the project’s objectives were achieved, and on providing evidence-based guidance for the sustainable integration of the self-help friendliness framework in healthcare. Patient-level outcomes, such as health literacy, quality of life, and self-management, were not assessed in accordance with agreements with the commissioning body. The aim of this study is to make the evaluation results accessible to the scientific community and provide evidence-based guidance for the sustainable integration of a self-help-friendly framework in healthcare systems.

## 2. Materials and Methods

**Figure 1:**
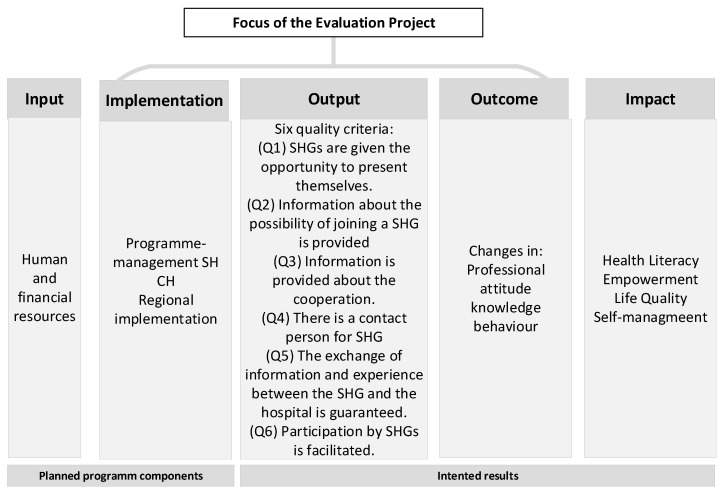
Logic model as basis for evaluation (simplified).

The evaluation was guided by a logic model [18], which made the relationships between inputs, outputs, and intended outcomes explicit and informed both the selection of indicators and the focus of data collection and analysis (see Figure 1). The model captured three main dimensions: (1) implementation, including the adoption of the self-help friendliness framework by participating hospitals and the establishment of cooperation teams with self-help centers and SHGs; (2) outputs, such as formalized agreements, defined measures, and institutionalized collaboration practices; and (3) outcomes, including changes in health professionals’ awareness of, attitudes toward, and engagement with SHGs as a complementary component of healthcare.

We developed the indicators and their corresponding measures at the outset of the evaluation in collaboration with Self-Help Switzerland. Subsequently, this project was approved by the commissioning body, Health Promotion Switzerland.

The mixed-methods evaluation design included document analyses, in-depth case studies, and a written survey to achieve methodological triangulation. Data collection was conducted annually between 2021 and 2024 (final cut-off: 31 October 2024).

During the study period, the three domains—implementation, output, and outcome—were assessed at national and regional levels. At the national level, the activities of Self-Help Switzerland were evaluated in relation to the project’s overall implementation, while regional-level analysis focused on the project’s implementation within four healthcare facilities. The following section outlines the methodological approach used to collect and analyze the data.

### 2.1. Sample

As part of this project, a multiplier approach was utilized to promote the dissemination and sustainable inclusion of self-help-friendly structures and practices into healthcare institutions. In this context, the multiplier approach involves the strategic engagement of individuals or institutions who disseminate health-related knowledge, skills, and strategies within their respective target groups [21]. 

In this evaluation, the multiplier approach was conceptualized as a cascading implementation and dissemination strategy aimed at integrating and ensuring the sustainability of the self-help-friendly framework within healthcare institutions involving two groups of multipliers. Multiplier 1 (M1) comprised cooperation team members, including professionals from self-help centers, hospital self-help coordinators, and representatives of SHGs. Multiplier 2 (M2) represented the target group of institution-specific measures, namely, healthcare professionals from the participating hospitals, named “hospital staff” within the project.

### 2.2. Document Analysis

This evaluation method involved a systematic review of predefined documents, including award dossiers, meeting protocols, training materials, and communication outputs (see Table 1). Self-Help Switzerland collected these documents from participating cooperation teams and then forwarded them on to us in our role as evaluators. We developed a structured coding framework and operationalized it using an Excel-based coding matrix. Document analysis was conducted using MAXQDA 24 and IBM SPSS Statistics 29.0.2.0 (20). To enhance analytical rigor and ensure intercoder reliability, all documents were independently coded by two team members, with discrepancies resolved through discussion.

### 2.3. Case Studies

To gain a deeper understanding of contextual factors, case studies were conducted at both national and regional levels. Detailed information on data sources, timing, and sample sizes is provided in Table 2. At the national level, data collection included annual interviews with members of the leadership team at Self-Help Switzerland. At the regional level, four cases of cooperation were explored through interviews with M1, complemented by observations of team meetings. An adaptive case selection strategy was employed, capturing different trajectories and settings for collaboration: a delayed implementation process (case 1), a rapidly initiated collaboration (case 2), a case in which collaboration ultimately failed to be realized (case 3), and a collaboration within the context of outpatient care (case 4) (see Appendix A).

### 2.4. Written Survey

To conduct the written survey, a hybrid design combining repeated cross-sectional baseline assessments with a longitudinal follow-up of an eligible sub-cohort was implemented. Baseline data (T0) were collected at the start of the collaboration of the respective cooperation teams, resulting in multiple entry cohorts over the course of the project. Follow-up data (T1) were collected approximately two years after the cooperations started. Given the rolling recruitment window during the four-year project, only individuals enrolled before October 2022 met the eligibility criterion for a two-year follow-up before the project ended in October 2024. Due to staff turnover in the participating hospitals, it was not possible to survey the same individuals at both measurement points. Consequently, a repeated between-group comparison was conducted over time to examine changes in knowledge, attitudes, and behaviors among members of the cooperation teams (M1) and among the broader group of healthcare professionals within the hospitals (M2). The same questionnaires were used at T0 and T1, with slight modifications applied only to questions tailored to the respective target groups (e.g., according to their role). Table 3 lists detailed information about the sample size.

The exact number of contacted M1 participants (members of the cooperation teams) was known, allowing the response rate to be calculated; however, the response rate of healthcare professionals in hospitals could not be determined.

The questionnaires for hospital staff were distributed via the designated self-help coordinators in each hospital. Internal communication channels, such as e-mails, information boards, or intranet postings, were used for dissemination.

To ensure methodological rigor, the survey items were assessed for content validity and face validity through iterative expert feedback. A pilot test was conducted to examine the clarity, relevance, and practical applicability of the questionnaire, which also provided initial indications of reliability in terms of consistent interpretation and response patterns across participants. Most items were single-item measures. This approach was chosen to ensure feasibility and minimize the burden on respondents, given the resource constraints and the practical demands of data collection in clinical settings. Consequently, internal consistency indices (e.g., Cronbach’s alpha) were not applicable; as such, measures require multi-item scales. Data were analyzed descriptively using SPSS Statistics 29.0.2.0 (20). No inferential statistical tests were conducted because the sample composition differed between T0 and T1, and complete panel data were not available.

### 2.5. Ethical Considerations

The study received ethical approval from the Ethics Committee of North and Central Switzerland. All participants were informed about the study’s aims, procedures, and data protection measures and provided written informed consent prior to participation. No patient-level data were collected at any point. Data collection and storage followed ethical guidelines for research involving human participants, ensuring confidentiality and the secure handling of all study materials.

## 3. Results

The results are presented according to the structure of the underlying logic model (see Figure 1).

### 3.1. Implementation

The results of the document analysis (see Table 1) show that the models’ implementation was uneven across linguistic regions. By the end of the evaluation period, a total of 56 cooperation agreements had been established nationwide, primarily in German-speaking regions (*k* = 48), with fewer implementations in French-speaking areas (*k* = 8), and none in the Italian-speaking part of Switzerland.

Certification and re-award data indicate that a substantial number of cooperations met the formal requirements of the self-help-friendliness label during the evaluation period. Overall, a total of 26 cooperations received a certificate because they fulfilled the six quality criteria of the self-help friendliness label. By the cut-off date (31 October 2024), 12 cooperations were eligible to apply for re-award. Of these, ten submitted their re-award documentation, which was assessed by Self-Help Switzerland, which verified the full and adequate implementation of SHGs. One cooperation was denied re-award after Self-Help Switzerland identified shortcomings in the implementation of measures during the review process, while another hospital did not submit its re-award documents, despite the two-year period having elapsed.

Findings from the four regional case studies provide further insights into the factors shaping the implementation process of cooperation agreements (see Table 2). Enabling factors were primarily identified at the relational, communicative, and organizational levels. The quality of interprofessional relationships between SHG members and healthcare professionals emerged as a key factor for a successful implementation process. In total, 87.5% of cooperation teams incorporated three or more disciplines. Cooperations were supported by dedicated self-help liaisons and anchored in formalized processes and practices, which are characterized by stable and sustained structures. Qualitative data further illustrate how repeated interaction fosters trust and mutual recognition over time:

*“And then I was allowed to come to the clinic once a month and talk about myself—about my story and about my group. And of course, I noticed… Even though it was always a different chief physician, they were always interested. And the longer it went on, the more interested they became.”*

*(SHG member, Case Study 1)*

Additional enabling factors included strategic communication measures such as testimonials, awareness campaigns, and participation in conferences, which increased visibility among decision-makers and supported their acceptance within the healthcare sector. The institutionalization of cooperation through clearly defined roles, responsibilities, and procedures also contributed to continuity and stability.

At the same time, the case studies identified several challenges to sustained implementation. Staff turnover and changes in leadership disrupted established relationships and working routines. A further challenge was the reliance on voluntary engagement for SHG members, whose participation could be limited by health-related circumstances or caregiving responsibilities. In addition, staff at the self-help center reported limited recognition and insufficient inclusion of SHG members within cooperation structures, which could weaken their continued engagement. One staff member from a self-help center described how an SHG member expressed frustration about the lack of appreciation they received and limited involvement in the project:

*“Up to a point, one of the self-help group members also says: if I receive so little appreciation, then I don’t want to continue doing this. I also think she is right in saying that she was not sufficiently considered in this project.”*

*(Self-help center, Case Study 4)*

Overall, the findings show that the implementation of cooperation agreements was shaped not only by formal agreements and certification processes, but also by the quality, continuity, and recognition of relationships between SHGs and healthcare professionals.

### 3.2. Output

The following section presents the results of the output evaluation, with particular emphasis on the six pre-established quality criteria.

*Quality Criterion 1: Enabling self-representation of self-help groups*

One of the fundamental prerequisites for effective collaboration is that SHGs are able to present themselves within healthcare institutions. An analysis of the catalog of measures document indicates that multiple measures have been implemented to enhance the visibility of SHGs in hospitals, including distributing flyers and posting information on screens or websites.

Written surveys among cooperation teams (M1) support these findings. As shown in Table 4, the perceived visibility of SHGs in hospitals improved from baseline to follow-up measurements across all respondent groups. In the total sample, overall agreement increased from 67.2% at baseline to 89.1% at follow-up, while “do not know” responses decreased from 10.3% to 1.8%. Similar trends were observed among SHGs, self-help centers, and hospital respondents.

*Quality Criterion 2: Providing information at the appropriate time*

Timely communication of opportunities to participate in SHGs is essential for enabling access to and engagement in SHGs. The quality criterion was assessed in the online survey within the cooperation team (M1). As shown in Table 5, perceptions of the availability of information and its impact on participation in SHGs improved from baseline to follow-up. Favorable responses increased overall, while uncertain and negative responses decreased. This pattern was observed consistently across all respondent groups.

*Quality Criterion 3: Internal communication regarding self-help cooperation*

Ensuring that hospital personnel are adequately informed about the collaboration between hospitals and SHGs is another essential element for effective implementation. Document analysis indicates that all 26 awarded cooperation projects met this criterion by providing such information within their institutions.

*Quality Criterion 4: Availability of a contact person for self-help*

A key factor in the effective and sustainable integration of self-help practices within healthcare institutions is the appointment of a clearly identifiable hospital staff member who acts as a coordinator and contact person for self-help centers. Among self-help coordinators, responses indicated full implementation of this criterion at both measurement points. At T0, 44 out of 45 participants either identified themselves as the contact person or referred to the designated person within their cooperation. At T1, 23 out of 24 participants likewise either identified themselves as the contact person or referred to the designated person in their institution. Thus, all cooperative teams had a defined contact person at both measurement points.

*Quality Criterion 5: Exchange of information and experiences*

Facilitating the exchange of information and lived experiences among SHGs, self-help centers, and hospitals is a key factor in promoting patient-centered care and developing tailored interventions. Table 6 shows perceptions on the exchange of information and experiences. The agreement was already high at baseline among the members of the cooperation teams and improved further at follow-up. Favorable responses increased overall, while neutral, negative, and “do not know” responses became rare or disappeared entirely at follow-up.

*Quality Criterion 6: Enabling self-help group participation*

Finally, ensuring that SHGs can participate actively within hospital structures is the sixth key element in the effective implementation of self-help friendliness measures. Table 7 indicates that the facilitation of participation by SHGs was rated positively by most respondents at both measurement points. Overall, favorable responses were slightly higher at follow-up, whereas negative and uncertain responses remained uncommon throughout. Additionally, document analysis and regional case studies demonstrated that a range of measures were implemented to support participation, including inviting SHGs to relevant public or internal training events and involving them in quality circles.

### 3.3. Outcome

The following section on outcomes examines changes in healthcare professionals’ knowledge, attitudes, and behaviors regarding the perception of self-help as a complementary component of inpatient care and aftercare. Data was collected via surveys distributed to hospital staff (M2).

Perception and institutional anchoring of self-help as a complementary component of hospital and aftercare services were operationalized using two indicators. The first indicator measured the proportion of hospital staff who perceived SHGs as a complementary offering to hospitalization. Table 8 shows that self-help was perceived as a complementary service to inpatient or outpatient treatment by almost 90% of respondents at both time points (the sum of “agree or somewhat agree” and “partly agree”). The respondents included hospital staff and self-help coordinators. Overall, favorable responses increased from baseline to follow-up, and the same trend was observed among both self-help coordinators and hospital staff.

The second indicator captured the proportion of hospital staff, which included hospital staff and self-help coordinators, who perceived SHGs as a complementary aftercare service. Table 9 indicates a more positive perception of self-help as an after-care service over time. Measures of agreement increased overall as well as within both respondent groups, with the clearest improvement observed among self-help coordinators.

The next outcome assesses whether information practices for patients and their relatives improved in self-help-friendly hospitals, particularly regarding the timely provision of information about available SHGs. As shown in Table 10, responses by hospital staff became more positive from baseline to follow-up, with an increase in favorable ratings and a percentage decline in negative and uncertain responses.

In parallel, the structural visibility of SHGs within hospitals increased. As shown in Table 11, the proportion of hospital staff reporting that a contact person for self-help groups existed increased from baseline to follow-up. Correspondingly, the proportion reporting that no such contact person existed decreased over time.

In addition to the outcomes reported for hospital staff (M2), a change in attitude within the cooperation team (M1) is highlighted. Cooperation on an equal footing, meaning that all team members have a voice and experience a balanced partnership, is crucial for self-help friendliness strategies. A further relevant finding, shown in Table 12, concerns attitudes within the cooperation team. Cooperation on an equal footing was rated positively overall at both measurement points, but subgroup differences were observed. At baseline, complete agreement was lower among the respondents of self-help centers than among SHG respondents and self-help coordinators. At follow-up, the proportion of SHG respondents reporting complete agreement was lower than that at baseline. By contrast, complete agreement among self-help coordinators remained high across both measurement points.

## 4. Discussion

Consistent with previous studies in Germany, the evaluation results suggest that integrating self-help friendliness within healthcare systems is feasible [22], Formalized cooperation structures may be linked to behavioral and cultural changes among hospital staff [11,23]. Staff members reported gradually incorporating cooperation and responsibilities into daily routines, informing patients about SHGs on a frequent basis, and perceiving SHGs as complementary to professional healthcare. However, these findings are based on descriptive data and should be interpreted cautiously.

The findings suggest that health professionals view SHGs as a complementary component of inpatient care and aftercare. They also see SHGs as a facilitator of cooperation among hospitals, self-help centers, and SHGs across Switzerland. The fact that 10 out of 12 eligible hospitals sought to renew the award and strong interprofessional representation was identified within the cooperation teams demonstrates the successful institutionalization of SHGs and cross-boundary collaboration. Awareness of designated contact persons extended beyond cooperation teams, suggesting that coordination structures were embedded in everyday practice.

In total, 90% of SHG representatives reported an improvement in project development. Moreover, the proportion of respondents from the cooperation teams who fully agreed that collaboration took place on an equal footing increased from baseline to follow-up. However, agreement declined among a subgroup of SHG respondents. These findings highlight persistent asymmetries in professional–lay collaboration and underscore the need for continuous reflection to ensure equitable cooperation. These findings highlight the importance of actively addressing power imbalances, ensuring that lay participants have meaningful opportunities to contribute to decision-making, and implementing targeted measures to promote equity and participation. Future initiatives should include structured feedback mechanisms, training for both professionals and SHG representatives, and monitoring of collaborative processes to ensure that the benefits of self-help involvement are experienced equitably across all stakeholders.

Two cross-cutting issues emerge. First, institutional volatility—such as staff turnover and leadership changes—undermined the continuity of collaboration in several hospitals. This finding highlights the need for stable, well-resourced structures and networks, standardized processes, and shared ownership that can endure personnel changes. Successful implementation requires designated self-help individuals who act as contacts to ensure continuity, coordination, and quality of support. Success also depends on the commitment of leadership members to securing resources and embedding the model within organizational structures and cultures. These two elements—professional responsibility and leadership commitment—are complementary and essential for sustainability.

Second, sustaining SHGs and volunteer engagement remains an ongoing challenge. In this evaluation, these challenges emerged both as newly identified issues and as recurring themes from previous studies in Germany. SHG members often reach the limits of their voluntary resources, especially because many of them live with one or more chronic conditions. Moreover, active involvement in healthcare institutions or committees requires skills that extend beyond lived experience alone [11,23,24]. Implementing the self-help-friendliness framework may therefore involve additional responsibilities, which can reduce the voluntary nature of participation. Previous research highlights the need to inform healthcare professionals about self-help, clarify responsibilities between staff and SHG members, and foster a deeper understanding of patient experiences and needs [11]. Because participation is voluntary and unpaid, sustained collaboration is difficult to achieve. Therefore, involving SHG members in quality circles and providing symbolic acknowledgment or financial recognition are crucial for maintaining engagement and long-term effectiveness.

### Strengths and Limitations of the Evaluation

Key strengths of this study include the evaluation’s national scope, the mixed-methods design, and prespecified, jointly agreed-upon indicators. These features enabled a clear analytical pathway for implementation, outputs, and outcomes, with triangulation across documents, written surveys, and case studies. This design supports confidence in the implementation results, the outputs of the six quality criteria, and the observed shifts in knowledge, attitudes, and behaviors identified among health professionals. Despite these strengths, several methodological limitations must be acknowledged.

The evaluation was designed to assess the structural and organizational conditions for integrating SHGs into hospital care, as well as resultant changes in healthcare professionals’ knowledge, attitudes, and behaviors. Consequently, this study does not allow conclusions regarding effects on patients or their relatives to be drawn. This distinction is important because, although SHG involvement is expected to support patient-centered care and health literacy, its impact on patient-reported outcomes, experiences, or health literacy remains unknown, as these were not assessed in the present study.

A major limitation of the study relates to the survey design and sampling. Due to staff turnover in the participating hospitals, the same individuals could not be surveyed at both time points. Consequently, a longitudinal design was not feasible, and a group comparison was conducted instead. Respondents were recruited via designated self-help coordinators, and recruitment strategies varied across institutions. As a result, the sample of hospital staff cannot be considered representative of the entire hospital, and the findings should be interpreted with caution.

Attrition between the two survey time points posed an additional methodological challenge. As a result of staff turnover, internal transfers, and rolling cohort entry, the data included non-equivalent samples that could not be linked at the individual level, and comparability across time points is limited. Moreover, the number of respondents was substantially higher at T0 than at T1.

In addition, no inferential statistical analyses were conducted. Given the changing sample composition and the predominantly single-item ordinal or categorical outcome measures, such analyses were limited in appropriateness and could have produced misleading results. Moreover, high compliance rates largely reflect the fact that participating hospital staff were already supportive of self-help, rather than representing statistically controlled effects. Therefore, the findings reflect descriptive trends within participating hospitals rather than statistically confirmed effects.

Furthermore, since the questionnaire for hospital staff was distributed through each hospital’s designated self-help coordinators, it was not possible to verify the extent to which hospital staff were informed about the survey. Consequently, the sample cannot be considered representative of the entire hospital staff.

Additional limitations include the potential inclusion of socially desirable responses and the absence of patient-level outcome measures.

While this study provides insights into implementation, outputs, and changes in attitudes at the professional level, including healthcare professionals’ knowledge and behaviors, it does not allow conclusions to be drawn about the impacts of self-help on patients or their relatives. Future studies should incorporate patient-level outcome measures to assess the project’s impact on health literacy, self-management, and quality of life, determining whether structural and procedural changes within hospitals translate into measurable benefits at both individual and system levels. Comparative analyses of healthcare professionals not involved in the project would also strengthen causal inference regarding attitude changes at the professional level. Finally, longitudinal designs are recommended to evaluate sustainability and long-term effects, rather than relying on cross-sectional group comparisons.

## 5. Conclusions

Two main implications arise from this evaluation.

First, the use of clear, auditable structures aligned with the six quality criteria of the self-help friendliness framework is a pragmatic approach that supports patient-centeredness and patient participation in hospitals, while minimizing procedural burdens.

Second, sustaining cooperative partnerships requires continuous facilitation, recognition, and clarity of roles. The slight decline in SHG members’ perceptions that collaboration takes place on an equal footing indicates that maintaining reciprocity over time demands active moderation, leadership commitment, and supportive structures to balance professional and voluntary contributions.

Overall, this study indicates that integrating patient-centeredness, participation, and structural collaboration into hospital settings is feasible and can be sustainable, provided it is guided by a clearly articulated self-help friendliness framework that includes defined quality criteria and a catalog of measures. The observed improvements in coordination and involvement of SHGs suggest that such an approach can effectively shape healthcare professionals’ knowledge, attitudes, and behaviors. Sustained success, however, depends on stable institutional structures, designated contact persons, and committed leadership. Equally important is the genuine recognition of SHG engagement as a form of lay expertise grounded in lived experience.

Taken together, these findings suggest that structured participation, coordination, and equitable collaboration are key mechanisms for advancing patient-centered care throughout the patient pathway. This study suggests that implementing the six quality criteria of the self-help friendliness framework can support the involvement of SHGs as a complementary service in healthcare.

## Figures and Tables

**Table 1:** Target groups and database of the document analysis.

Target Group	Database
Members of cooperation teams (M1)	Documents from 56 cooperations, including 45 cooperations with a catalog of measures, which were developed specifically for the institution based on the six quality criteria 26 cooperations (of the 56) with award documents, including catalog of measures, protocols of team meetings (M1) for the self-evaluation of implementation, and an explanation by the local project manager from the self-help center about how SHGs were integrated into the cooperation team 10 cooperations (of the 56) with re-award documents (new award documents) and 3 with second re-award documents
National project management	Project documents from Self-Help Switzerland (work aids, protocols, review documents, annual reports, training, and communication concepts)

**Table 2:** Overview of data sources, timing, and sample sizes for the national and regional case studies (T0–T1).

Study Component	Data Source	Method	Timing/ Frequency	Participants/ Sample Size
National case study	SH CH project management	Interview	Annually	Project management: *n* = 1
	Working group	Observation	Twice a year	*n* = 8–12 participants per meeting
	Experience-exchange meetings	Observation	Twice a year	On average, 23 participants per meeting; 5 meetings during the evaluation period
Regional case studies	Case 1	Interviews and meeting observations	T0: Year 2 T1: Year 4	SHC: *n* = 1 SHG: *n* = 2 self-help coordinators: *n* = 1 hospital staff: *n* = 3
	Case 2		T0: Year 2 T1: Year 4	SHC: *n* = 1 SHG: *n* = 1 self-help coordinators: *n* = 1 hospital staff: *n* = 3
	Case 3		T0: Year 3 T1: Year 4	SHC: *n* = 1 self-help coordinators: *n* = 1
	Case 4		T0: Year 3 T1: Year 4	SHC: *n* = 1, SHG: *n* = 2, self-help coordinators: *n* = 1

Abbreviations: SHC, self-help centers; SH CH, Self-Help Switzerland; SHG, self-help groups; T0 = first time interviewed in the corresponding evaluation year; T1 = second time interviewed in the corresponding evaluation year.

**Table 3:** Written survey sample sizes at measurement points T0 and T1 by participant group.

Participant Group	T0, *n*	T1, *n*
**Cooperation teams (M1)**		
Self-help centers participants	40	21
Self-help groups participants	46	10
Self-help coordinators	45	24
**hospital specialists (M2)**		
Hospital staff	399	226
**Total**	530	281

Notes: T0 = measurement point 1 (baseline); T1 = measurement point 2 (~2 years after T0; follow-up).

**Table 4:** Perceived visibility of self-help and/or self-help groups in the hospital at T0 and T1 according to the respondent group.

Measurement Point	Respondent Group	Valid/Missing, *n*	Agree or Somewhat Agree, *n* (%)	Partly Agree, *n* (%)	Somewhat Disagree or Disagree, *n* (%)	Do Not Know, *n* (%)
T0	**Cooperation teams**	**116/15**	**78 (67.2)**	**22 (19.0)**	**4 (3.4)**	**12 (10.3)**
	Self-help groups participants	37/9	23 (62.2)	6 (16.2)	2 (5.4)	6 (16.2)
	Self-help centers participants	39/1	25 (64.1)	8 (20.5)	2 (5.1)	4 (10.3)
	Self-help coordinators	40/5	30 (75.0)	8 (20.0)	1 (2.5)	1 (2.5)
T1	**Cooperation teams**	**55/0**	**49 (89.1)**	**5 (9.1)**	**0 (0.0)**	**1 (1.8)**
	Self-help groups participants	10/0	7 (70.0)	2 (20.0)	0 (0.0)	1 (10.0)
	Self-help centers participants	21/0	20 (95.2)	1 (4.8)	0 (0.0)	0 (0.0)
	Self-help coordinators	24/0	22 (91.7)	2 (8.3)	0 (0.0)	0 (0.0)

Notes: T0 = measurement point 1 (baseline); T1 = measurement point 2 (~2 years after T0; follow-up). Percentages are based on valid responses.

**Table 5:** Providing information at the appropriate time for participation in self-help groups: responses at T0 and T1 from the respondent group.

Measurement Point	Respondent Group	Valid/Missing, *n*	Agree or Somewhat Agree, *n* (%)	Partly Agree, *n* (%)	Somewhat Disagree or Disagree, *n* (%)	Do Not Know, *n* (%)
T0	**Cooperation teams**	**116/18**	**70 (60.3)**	**20 (17.2)**	**9 (7.8)**	**17 (14.7)**
	Self-help groups participants	36/12	21 (58.3)	6 (16.7)	1 (2.8)	8 (22.2)
	Self-help centers participants	39/1	21 (53.8)	7 (17.9)	2 (5.1)	9 (23.1)
	Self-help coordinators	41/5	28 (68.3)	7 (17.1)	6 (14.6)	0 (0.0)
T1	**Cooperation teams**	**55/0**	**46 (83.6)**	**5 (9.1)**	**0 (0.0)**	**4 (7.3)**
	Self-help groups participants	10/0	7 (70.0)	1 (10.0)	0 (0.0)	2 (20.0)
	Self-help centers participants	21/0	19 (90.5)	2 (9.5)	0 (0.0)	0 (0.0)
	Self-help coordinators	24/0	20 (83.3)	2 (8.3)	0 (0.0)	2 (8.3)

Notes: T0 = measurement point 1 (baseline); T1 = measurement point 2 (~2 years after T0; follow-up). Percentages are based on valid responses.

**Table 6:** Exchange of information and experiences: responses of cooperation team members at T0 and T1 from the respondent group.

Measurement Point	Respondent Group	Valid/Missing, *n*	Agree or Somewhat Agree, *n* (%)	Partly Agree, *n* (%)	Somewhat Disagree or Disagree, *n* (%)	Do Not Know, *n* (%)
T0	**Cooperation teams**	**116/18**	**103 (88.8)**	**5 (4.3)**	**3 (2.6)**	**5 (4.3)**
	Self-help groups participants	36/12	32 (88.9)	2 (5.6)	1 (2.8)	1 (2.8)
	Self-help centers participants	39/1	33 (84.6)	2 (5.1)	1 (2.6)	3 (7.7)
	Self-help coordinators	41/5	38 (92.7)	1 (2.4)	1 (2.4)	1 (2.4)
T1	**Cooperation teams**	**55/0**	**54 (98.2)**	**1 (1.8)**	**0 (0.0)**	**0 (0.0)**
	Self-help groups participants	10/0	9 (90.0)	1 (10.0)	0 (0.0)	0 (0.0)
	Self-help centers participants	21/0	21 (100.0)	0 (0.0)	0 (0.0)	0 (0.0)
	Self-help coordinators	24/0	24 (100.0)	0 (0.0)	0 (0.0)	0 (0.0)

Notes: T0 = measurement point 1 (baseline); T1 = measurement point 2 (~2 years after T0; follow-up). Percentages are based on valid responses.

**Table 7:** Participation of SHGs is enabled: responses of cooperation team members at T0 and T1 from the respondent group.

Measurement Point	Respondent Group	Valid/Missing, *n*	Agree or Somewhat Agree, *n* (%)	Partly Agree, *n* (%)	Somewhat Disagree or Disagree, *n* (%)	Do Not Know, *n* (%)
T0	**Cooperation teams**	**114/17**	**96 (84.2)**	**8 (7.0)**	**4 (3.5)**	**6 (5.3)**
	Self-help groups participants	36/12	33 (91.7)	1 (2.8)	1 (2.8)	1 (2.8)
	Self-help centers participants	39/1	29 (74.4)	5 (12.8)	2 (5.1)	3 (7.7)
	Self-help coordinators	41/5	36 (87.8)	2 (4.9)	1 (2.4)	2 (4.9)
T1	**Cooperation teams**	**55/0**	**49 (89.1)**	**4 (7.3)**	**1 (1.8)**	**1 (1.8)**
	Self-help groups participants	10/0	10 (100.0)	0 (0.0)	0 (0.0)	0 (0.0)
	Self-help centers participants	21/0	18 (85.7)	2 (9.5)	1 (4.8)	0 (0.0)
	Self-help coordinators	24/0	21 (87.5)	2 (8.3)	0 (0.0)	1 (4.2)

Notes: T0 = measurement point 1 (baseline); T1 = measurement point 2 (~2 years after T0; follow-up). Percentages are based on valid responses.

**Table 8:** Self-help is perceived as a complementary service to inpatient or outpatient treatment: responses at T0 and T1 from the respondent group.

Measurement Point	Respondent Group	Valid/Missing, *n*	Agree or Somewhat Agree, *n* (%)	Partly Agree, *n* (%)	Somewhat Disagree or Disagree, *n* (%)	Do Not Know, *n* (%)
T0	**Total**	**429/15**	**320 (74.6)**	**57 (13.3)**	**31 (7.2)**	**21 (4.9)**
	Self-help coordinators	40/5	27 (67.5)	9 (22.5)	4 (10.0)	0 (0.0)
	Hospital staff	389/10	293 (75.3)	48 (12.3)	27 (6.9)	21 (5.4)
T1	**Total**	**250/0**	**202 (80.8)**	**21 (8.4)**	**17 (6.8)**	**10 (4.0)**
	Self-help coordinators	24/0	19 (79.2)	3 (12.5)	1 (4.2)	1 (4.2)
	Hospital staff	226/0	183 (81.0)	18 (8.0)	16 (7.1)	9 (4.0)

Notes: T0 = measurement point 1 (baseline); T1 = measurement point 2 (~2 years after T0; follow-up). Percentages are based on valid responses.

**Table 9:** Self-help is perceived as an aftercare service: responses at T0 and T1 from the respondent group.

Measurement Point	Respondent Group	Valid/Missing, *n*	Agree or Somewhat Agree, *n* (%)	Partly Agree, *n* (%)	Somewhat Disagree or Disagree, *n* (%)	Do Not Know, *n* (%)
T0	**Total**	**429/15**	**284 (66.2)**	**84 (19.6)**	**37 (8.6)**	**24 (5.6)**
	Self-help coordinators	40/5	24 (60.0)	11 (27.5)	5 (12.5)	0 (0.0)
	Hospital staff	389/10	260 (66.8)	73 (18.8)	32 (8.2)	24 (6.2)
T1	**Total**	**250/0**	**180 (72.0)**	**42 (16.8)**	**14 (5.6)**	**14 (5.6)**
	Self-help coordinators	24/0	20 (83.3)	2 (8.3)	1 (4.2)	1 (4.2)
	Hospital staff	226/0	160 (70.8)	40 (17.7)	13 (5.8)	13 (5.8)

Notes: T0 = measurement point 1 (baseline); T1 = measurement point 2 (~2 years after T0; follow-up). Percentages are based on valid responses.

**Table 10:** Patients and their relatives are sufficiently informed about the opportunity to participate in a self-help group: responses of hospital staff (M2) at T0 and T1.

Measurement Point	Valid/Missing, *n*	Agree or Somewhat Agree, *n* (%)	Partly Agree, *n* (%)	Somewhat Disagree or Disagree, *n* (%)	Do Not Know, *n* (%)
T0	389/10	161 (41.4)	91 (23.4)	81 (20.8)	56 (14.4)
T1	226/0	120 (53.1)	57 (25.2)	25 (11.1)	24 (10.6)

Notes: T0 = measurement point 1 (baseline); T1 = measurement point 2 (~2 years after T0; follow-up). Percentages are based on valid responses.

**Table 11:** Hospital staff (M2) awareness of a well-known contact person for self-help groups at T0 and T1.

Measurement Point	Valid/Missing, *n*	Yes, *n* (%)	No, *n* (%)
T0	399/0	180 (45.1)	219 (54.9)
T1	226/0	130 (57.5)	96 (42.5)

Notes: T0 = measurement point 1 (baseline); T1 = measurement point 2 (~2 years after T0; follow-up). Percentages are based on valid responses.

**Table 12:** Collaboration within the cooperation team on an equal footing: responses of cooperation team members at T0 and T1 from the respondent group.

Measurement Point	Respondent Group	Valid/Missing, *n*	Agree, *n* (%)	Somewhat Agree, *n* (%)	Partly Agree, *n* (%)	Somewhat Disagree or Disagree, *n* (%)	Do Not Know, *n* (%)
T0	**Cooperation teams**	114/17	91 (79.8)	17 (14.9)	3 (2.6)	1 (0.9)	2 (1.8)
	Self-help groups participants	35/11	30 (85.7)	4 (11.4)	0 (0.0)	1 (2.9)	0 (0.0)
	Self-help centers participants	39/1	25 (64.1)	10 (25.6)	2 (5.1)	0 (0.0)	2 (5.1)
	Self-help coordinators	40/5	36 (90.0)	3 (7.5)	1 (2.5)	0 (0.0)	0 (0.0)
T1	**Cooperation teams**	55/0	46 (83.6)	8 (14.5)	1 (1.8)	0 (0.0)	0 (0.0)
	Self-help groups participants	10/0	7 (70.0)	3 (30.0)	0 (0.0)	0 (0.0)	0 (0.0)
	Self-help centers participants	21/0	17 (81.0)	3 (14.3)	1 (4.8)	0 (0.0)	0 (0.0)
	Self-help coordinators	24/0	22 (91.7)	2 (8.3)	0 (0.0)	0 (0.0)	0 (0.0)

Notes: T0 = measurement point 1 (baseline); T1 = measurement point 2 (~2 years after T0; follow-up). Percentages are based on valid responses. For this item, the positive response categories are displayed separately because responses were strongly concentrated in the favorable range, and combining them would have obscured meaningful variations in results.

## Data Availability

The data presented in this study are available on request from the corresponding author due to privacy restrictions. Raw interview data cannot be shared to protect participant confidentiality; however, aggregated data will be provided upon reasonable request.

## Abbreviations

The following abbreviations are used in this manuscript:

M1	Multiplier 1 (members of cooperation teams: self-help-center participants, self-help-group participants, and self-help coordinators)
M2	Multiplier 2 (hospital staff)
MAXQDA	Qualitative data analysis software
SHC	Self-help center
SH CH	Self-Help Switzerland
SHF	Self-help friendliness framework
SHGs	Self-help group
SPSS	Statistical Package for the Social Sciences
T0	Baseline measurement
T1	Follow-up measurement

## Appendix A

**Table A1:** Characteristics of the four case studies (cases 1–4).

Characteristic	Case 1: Slow Start of Cooperation	Case 2: Rapid Cooperation	Case 3: Case That Did Not Come to Fruition	Case 4: Outpatient Setting
Starting year	2021	2021	-	2021
Award year	2024	2022	-	2023
Area of health	Mental health	Dermatology	Mental health	Mental health
Number of SHG members in cooperation team	2	1	-	2

Abbreviations: SHG, self-help group. Note: “-” indicates not applicable/not available (the case did not come to fruition).
